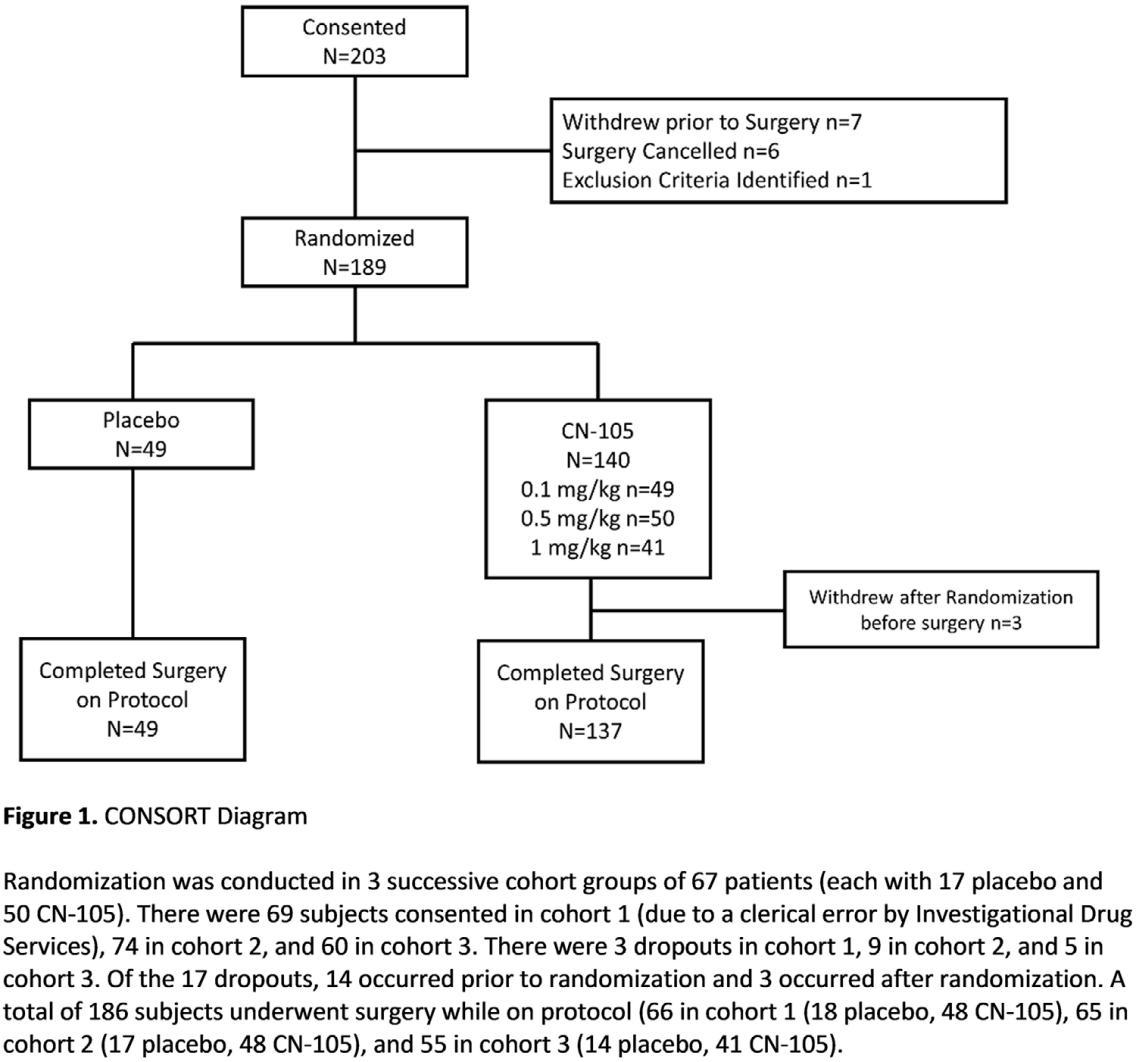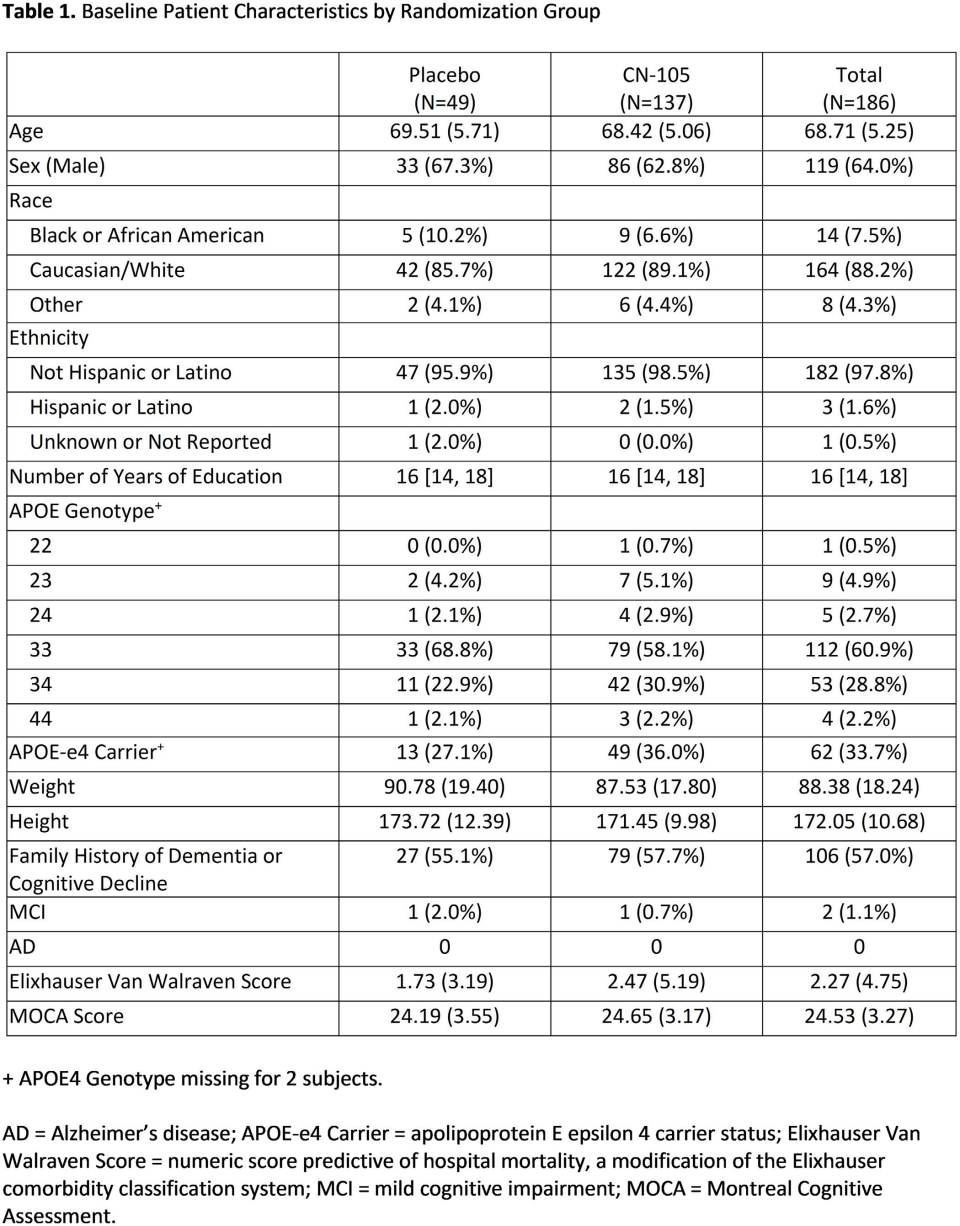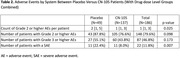# The Safety, Feasibility and Effect of the APOE Mimetic Peptide CN‐105 on Neuroinflammation and Delirium after Major Non‐cardiac/Non‐neurologic Surgery in Older Adults: Results from the Phase II, Triple Blind, Escalating Dose, MARBLE Randomized Clinical Trial

**DOI:** 10.1002/alz.095338

**Published:** 2025-01-09

**Authors:** Miles Berger, Noah J. Timko, Piper C. Boykin, Jacqueline M. Emerson, Marty G. Woldorff, Mary Cooter Wright, J. Taylor Herbert, Eugene W. Moretti, Michael J. Devinney, David Ryu, Keith W. VanDusen, Thomas Bunning, Bethany J. Hsia, Jeffrey N. Browndyke, Kaj Blennow, Henrik Zetterberg, Daniel T. Laskowitz, the MARBLE Study Investigators

**Affiliations:** ^1^ Anesthesiology Department, Duke University School of Medicine, Durham, NC USA; ^2^ Duke/UNC Alzheimer’s Disease Research Center, Duke University School of Medicine, Durham, NC USA; ^3^ Center for the Study of Aging & Human Development, Duke University, Durham, NC USA; ^4^ Center for Cognitive Neuroscience & Duke Institute for Brain Sciences (DIBS), Duke University, Durham, NC USA; ^5^ Duke University School of Medicine, Durham, NC USA; ^6^ Department of Psychiatry and Behavioral Sciences, Duke University Medical Center, Durham, NC USA; ^7^ Durham VA Medical Center, Durham, NC USA; ^8^ University of Gothenburg, Gothenburg Sweden; ^9^ Neurology Department, Duke University, Durham, NC USA

## Abstract

**Background:**

APOE4 leads to increased neuroinflammation, neurocognitive decline, increased risk of Alzheimer’s disease, and may be associated with increased delirium risk. However, the safety and feasibility of pharmacologic modulation of APOE to prevent neuroinflammation and postoperative delirium is unclear.

**Methods:**

We performed a Phase II, triple blind, escalating dose, randomized controlled trial to determine the safety, feasibility, and efficacy of the APOE mimetic peptide CN‐105 for preventing postoperative neuroinflammation and delirium. We enrolled older adults (age ≥ 60 years) undergoing major non‐cardiac/non‐neurologic surgery with a planned overnight hospital stay. Patients were randomized 1:3 to placebo or intravenous CN‐105 (0.1, 0.5 or 1 mg/kg) starting within 1 hour before surgery and continuing every 6 hours (± 90 minutes) afterwards, until 13 doses were received or hospital discharge (whichever occurred first). The primary outcome was the number of Grade 2 or higher adverse events (AEs) among patients randomized to CN‐105 vs. placebo. Secondary outcomes included percent of drug doses administered within the correct time window, and postoperative delirium incidence and severity.

**Results:**

203 patients were enrolled; 186 patients (mean [SD] age, 68.7 [5.3] years; 119 male) completed surgery on protocol (49 [26%] Placebo, 137 [74%] CN‐105; Figure 1). The groups were generally well‐matched (Table 1). Patients administered CN‐105 vs. placebo had less Grade 2 or higher AEs (median per patient [Q1, Q3] 1 [1, 3] vs. 2 [1, 5]; p = 0.025), and a lower rate of severe AEs (8.0% vs. 22.4%, p = 0.007; Table 2). The rate of doses administered within window was 95.3% (95% CI: 93.7%‐96.6%) for CN‐105‐treated patients and 93.8% (95% CI: 90.8%‐96.0%) for placebo‐treated patients. CN‐105 (vs. placebo) treated patients had a lower delirium incidence (19.3% vs 27.1%, relative risk = 0.71 [95% CI: 0.40‐1.27], p = 0.26), and lower median delirium severity (1 vs. 2; proportional odds ratio [95% CI] 0.66 [0.37, 1.20]; p = 0.178), though these effects were not statistically significant.

**Conclusions:**

CN‐105 is safe and feasible to administer to older non‐cardiac/non‐neurologic surgery patients, and led to fewer postoperative Grade 2 or higher AEs and severe AEs. The 95% CIs for the effects of CN‐105 included clinically relevant reductions in delirium incidence and severity.